# IFN-I Independent Antiviral Immune Response to Vesicular Stomatitis Virus Challenge in Mouse Brain

**DOI:** 10.3390/vaccines8020326

**Published:** 2020-06-19

**Authors:** Anurag R. Mishra, Siddappa N. Byrareddy, Debasis Nayak

**Affiliations:** 1 Discipline of Biosciences and Biomedical Engineering, Indian Institute of Technology Indore, Indore, MP 453 552, India; phd1501271012@iiti.ac.in; 2 Department of Pharmacology and Experimental Neuroscience, University of Nebraska Medical Center, Omaha, NE 68198, USA

**Keywords:** CNS, VSV, TNF-α, IFN independent pathway, ISG expression, innate immune response

## Abstract

Type I interferon (IFN-I) plays a pivotal role during viral infection response in the central nervous system (CNS). The IFN-I can orchestrate and regulate most of the innate immune gene expression and myeloid cell dynamics following a noncytopathic virus infection. However, the role of IFN-I in the CNS against viral encephalitis is not entirely clear. Here we have implemented the combination of global differential gene expression profiling followed by bioinformatics analysis to decipher the CNS immune response in the presence and absence of the IFN-I signaling. We observed that vesicular stomatitis virus (VSV) infection induced 281 gene changes in wild-type (WT) mice primarily associated with IFN-I signaling. This was accompanied by an increase in antiviral response through leukocyte vascular patrolling and leukocyte influx along with the expression of potent antiviral factors. Surprisingly, in the absence of the IFN-I signaling (IFNAR^−/−^ mice), a significantly higher (1357) number of genes showed differential expression compared to the WT mice. Critical candidates such as IFN-γ, CCL5, CXCL10, and IRF1, which are responsible for the recruitment of the patrolling leukocytes, are also upregulated in the absence of IFN-I signaling. The computational network analysis suggests the presence of the IFN-I independent pathway that compensates for the lack of IFN-I signaling in the brain. The analysis shows that TNF-α is connected maximally to the networked candidates, thus emerging as a key regulator of gene expression and recruitment of myeloid cells to mount antiviral action. This pathway could potentiate IFN-γ release; thereby, synergistically activating IRF1-dependent ISG expression and antiviral response.

## 1. Introduction

The central nervous system (CNS) is equipped with a dynamic immunological response mechanism to deal with invading infection and injuries [1,2]. For example, specialized innate sentinels such as astrocytes and microglia can initiate a robust innate immune response during injury and infection [3,4,5]. These cells are equipped with innate sensing mechanisms (e.g., pattern recognition) to detect injury and invading pathogens, which in turn pave the pathway for an innate immune response marked by local cytokine secretion. This often leads to the recruitment of peripheral immune cells to the injury site. However, in normal physiological conditions, immune cell migration to the CNS is tightly regulated by the blood–brain barrier (BBB) [6] and blood–cerebral spinal fluid (CSF) barrier. Synergistically these mechanisms provide an extra layer of protection to the CNS, not only by restricting immune cells but also by preventing the entry of invading pathogens [7]. However, some pathogens still circumvent this defense, penetrate the BBB, and infect the CNS. A sizeable number of neurotropic viruses can get access to the CNS [8] via (a) inflammation-induced breakdown of BBB, (b) viral spread through the infected endothelial cells or peripheral nervous system, and (c) intracellular transport through incoming myeloid cells [9]. The consequence of CNS-pathogen interaction is manifested by various neurological sequels such as meningitis, encephalitis, etc. This pathogenesis either gets resolved without any apparent damage to the CNS or leads to persistent impairment of the brain functions, or occasionally, results in a fatality. 

Virus-induced CNS inflammation primarily manifests in the form of either meningitis or encephalitis. In the simplest term, meningitis refers to the inflammation of meninges (membrane lining below the skull), while encephalitis refers to inflammation of brain tissue. In clinical terms, the “aseptic meningitis” refers to inflammation where the etiological agent is non-bacterial in nature [9] that primarily results from a viral infection such as human enteroviruses (HEV) [10,11], St. Louis encephalitis virus (SLEV) [12], Herpes simplex virus (HSV) 1 and 2 [13], human immunodeficiency virus (HIV-1) [14], mumps virus [15], bunyaviruses [16,17,18], and lymphocytic choriomeningitis virus (LCMV). By using various animal models combined with in vitro studies, we now have a fair understanding of the mechanism of CNS pathogenesis during viral meningitis. For example, in the case of a non-cytopathic virus, it is well established that it is the host-induced immunopathology that leads to fatal meningitis in the immune-competent host. LCMV replication in the meninges induces secretion of inflammatory cytokines that lead to the massive recruitment of peripheral myeloid cells to the meningeal vasculature followed by vascular leakage and fatality to the host [19]. Here, type I interferon (IFN-I) signaling is the sole regulator of innate immune gene expression and myeloid cell dynamics [20]. Mice deficient with interferon-alpha receptor (*Ifnar*^−/−^) could not develop any meaningful antiviral response to LCMV challenge, which exposed a loophole in the brain’s defense mechanism. 

However, in the case of a cytopathic virus such as in case of encephalitis (e.g., rabies virus), rapid viral replication followed by the accumulation of apoptotic cells brings an additional challenge to the CNS to resolve the inflammation. These encephalitis-causing viruses include alphaviruses, flaviviruses, Japanese encephalitis virus (JEV), HSV1 [21], measles virus [22], rabies virus (RV) [23], VSV, and Chandipura virus (CHPV), etc., which are cytopathic in nature. A recent study identified that the CNS exerts strong cytokines and chemokines-mediated antiviral response to the cytopathic alphavirus (Chikungunya virus) through secretion of IFN-I, IFN-II, TNF, and antiviral NK cells [24]. Similarly, in another alphavirus, Sindbis virus infection, the Interferon-gamma (IFN-γ) modulates resolution of CNS inflammation by promoting tissue-resident CD8 memory T (T_RM_) response while restricting peripheral T cell entry [25]. In a similar line, West Nile fever virus (WNV) infection in a mouse model showed overexpression of proinflammatory chemokines such as CCL2, CCL5, and CXCL10, collectively modulating the immunopathology in the brain [26]. Other than IFN-I, various factors determine the quality of CNS immune response. These include the biological nature of viruses, entry mechanism and target cells in the brain, and host immune status etc. For example, in JEV infection, studies have shown that CX_3_CR1^+^CD11c^+^ DCs play a central role in activating NK cells and generating effective immune response [27].

In contrast to the notion of the protective role of IFN-I, studies conducted in Zika virus-induced encephalitis in mouse brain showed that IFN-I is insufficient to prevent the establishment of infection in mouse brain through innate immune response, rather adaptive response plays a major role in immunopathology [28]. Apart from innate responses to the pathogens, IFN-I plays an essential role in the development of CNS. Thus, dissection of IFN-I response in the brain is still required to understand the mechanistic insight into the IFN-I mediated response. To shed light in this direction, in this study, we compared the nature of the immune response to cytopathic virus infection in the presence and absence of IFN-I signaling. Using a murine model of VSV encephalitis, we assessed the CNS innate immune response by analyzing the global gene expression pattern, and myeloid cell dynamics in virally infected mouse brain.

The VSV is neurotropic and causes rapid induction of the programmed cell death pathway in infected cells. It is also highly sensitive to IFN-I induced antiviral response. Whole-brain transcriptome analysis showed that the CNS responded strongly to viral challenge through massive changes in gene expression, primarily mediated by IFN-I signaling. The IFN-I signaling pathway topped the list of canonical pathways followed by activation of interferon regulatory factors (IRF), pattern recognition, Retinoic acid-mediated apoptosis, etc. Intravital imaging, as well as flow cytometric analysis of VSV-infected brain, further revealed a massive influx of myelomonocytic cells and increased vascular patrolling of neutrophils. However, in the absence of IFN-I signaling, surprisingly, CNS could maintain an effective antiviral response through an alternate mechanism. More surprisingly, a significantly higher number of genes associated with olfactory function were activated in *Ifnar*^−/−^ mice compared to the wild type counterpart. Bioinformatics analysis further revealed the central role of IFNγ and tumor necrosis factor (TNF)-mediated immune responses, potentially activating the interferon-stimulated gene (ISG) expression to maintain an antiviral state. Our analysis suggests that *Ifnar*^−/−^ mice exerted the antiviral gene expression through IRF1-mediated pathways. 

## 2. Materials and Methods 

### 2.1. Animal Experimentation

Eight-week-old C57BL/6 (B6) and B6 IFN-I receptor knock-out (*Ifnar*^−/−^) female mice were used in the experiments. B6 mice were procured from the Jackson Laboratory, Bar Harbor, ME, USA, and B6 *Ifnar*^−/−^ were generously provided by Dr. Jonathan Sprent (formerly from The Scripps Research Institute). In accordance with approved protocols, the experimental procedures were performed under anesthesia and conducted in a manner to minimize animal suffering. Similarly, green fluorescence protein (GFP) expressing LysM-GFP reporter mice, heterozygous knock-in mice expressing GFP under the promoter of lysozyme gene of mice (LysM^gfp/+^) [29], were used to track neutrophil and monocyte population in the brain. All mice were bred and housed at the NIH animal facilities. 

### 2.2. Animal Ethics Statement

All mice in this study were handled in accordance with the guidelines set forth by the NIH Animal Care and Use Committee and the recommendations in the AAALAC Guide for the Care and Use of Laboratory Animals. The protocol was approved by the NINDS Animal Care and Use Committee (protocol # 1295-12).

### 2.3. Virus and Infections

The VSV Indiana strain (VSV_I_) was generously provided by Dr. Asit Pattnaik (University of Nebraska-Lincoln). The viral stocks were prepared using the low (0.1) multiplicity of infection (MOI) passaged in the BHK 21 cell line. The supernatant containing the virus was harvested around 24 h post-infection coinciding with the appearance of rounded apoptotic cells. Stock concentration was confirmed by using standard plaque assay method in Vero cells. For animal experiments, quantified plaque-forming units (PFU) of virus in 20 μL volume was injected intracerebrally into mice under anesthetic conditions. We initially attempted to do intranasal VSV inoculation for these experiments. However, the intracerebral route of inoculation showed consistency in the kinetics of pathogenesis and onset of neurological manifestation, and was hence chosen for the route of inoculation for the entire study.

### 2.4. Real-Time PCR

Infected mice were intracardially perfused with 25 mL of saline, and brains from the infected mice were extracted aseptically. The brains were homogenized in the 1 mL of RPMI media. The homogenized solution was centrifuged at 16,000× *g* for 5 min at 4 °C. The pellet fraction was used for the total cellular RNA isolation; whereas, the supernatant fraction was used for viral titer determination. The pelleted fraction was treated with Trizol (Invitrogen, German Town, MD, USA), and total RNA was extracted using the PureLink RNA mini kit (Invitrogen, USA). A total of 1 µg of RNA was treated with DNAse I (Invitrogen) and reverse transcribed using the iScript (Bio-Rad, Portland, ME, USA) with a mixture of random hexamer and oligo (dT) primers. Subsequently, RT-qPCR was performed in 96-well plates using the SoFast EvaGreen supermix (BioRad, Portland, ME, USA) in 20 µL volume reaction. The gene-specific primers (see Table S2) were used to determine relative gene expression where the housekeeping beta-actin gene was used for normalization data in all qPCR experiments. The qPCR reactions were performed in triplicates, and samples without treatment with reverse transcriptase were used as non-template control. The qPCR cycling condition consists of the initial denaturation at 95 °C for 3 min, followed by 40 cycles with denaturation at 95 °C for 10 s, gene-specific primer annealing at 58 °C for 10 s, and polymerase extension at 72 °C for 20 s.

### 2.5. Microarray Hybridization and Data Analyses

Gene microarray analysis experiments were conducted in C57BL/6 (B6) and B6 IFN-I receptor knock-out (*Ifnar*^6^) mice with a sample size of four animals (*n* = 4). First, day three post-infected mice brain tissue was collected aseptically after intracardiac perfusion of mice with 25 mL of saline. Subsequently, total cellular RNA was extracted, as mentioned above. For hybridizations, samples were prepared according to the Affymetrix protocol (Affymetrix, Inc., Sunnyvale, CA, USA). Further, the RNA quality and quantity were ensured using the bioanalyzer (Agilent, Inc., Santa Clara, CA, USA) and nanodrop (Thermo Scientific, Inc., Franklin, MA, USA), respectively. The microarray hybridization and analyses of samples were performed as per the Affymetrix protocol. The raw data ware curated by using various statistical tools such as Tukey box plot and principal component analysis (PCA), and scatter-plot analysis. The data visualization was done by correlation-based heat map analysis.

### 2.6. Intravital Imaging Experiment

Two-photon imaging was performed in Leica SP5 two-photon microscope (Leica, Wetzlar, Germany) equipped with an 8000-Hz resonant scanner, a 20X/1.0 NA water-dipping objective, and a Mai Tai HP DeepSee Laser (Spectra-Physics, Santa Clara, CA, USA) tuned to 920 nm. The brain imaging experiment was conducted on day three post-infection. During the entire period of imaging experiments, mice were kept anesthetized and placed on a hot pad at 37 °C. The skull bones were thinned manually, and the animal head was fixed on the platform to allow the microscope objective to collect visuals as previously described protocol by Yang et al. [30]. For visualization of blood vessels, 5 µL Qtracker non-targeted quantum dots (655 nm; 0.2 µm; Invitrogen) was injected intravenously in LysM^gfp/+^ mice challenged with 10^4^ PFU of VSV_I_ strain, which were imaged to visualize the presence of neutrophils, monocytes, and macrophages in the infected brain. The images were acquired by tuning the LASER at 920 nm, and imaging stacks were collected at 10–20 s intervals keeping stack step size at 2.5 µM. The collected images were further processed using IMARIS (Bitplane, Belfast, UK) for 3D and 2D construction. 

### 2.7. Flow Cytometry

The infected mouse brain was perfused with normal saline under anesthesia, and the brain was then collected and homogenized. The homogenized tissue was then digested in Collagenase-D in RPMI media at 37 °C for 30 min. The brain tissue was further homogenized by passing through a 100 µM cell strainer to make an individual cell suspension. The cell suspension was centrifuged at 1500 g for 30 min in the Ficol density gradient method to collect leukocytes. The leukocytes were then resuspended with Fc block solution for 10 min and subsequently stained with specific antibodies, as mentioned below. The data acquisition was conducted in LSR-II (BD Bioscience, San Jose, CA, USA) system, and the data analysis was done in FlowJo software (Tree Star, V 9.0, Ashland, OR, USA). Gating strategy for microglia was taken as follows; CD45^low^, Thy.12^−^, Gr1^−^, and CD11b^+^ population; for neutrophils, gating was based on CD45^low^, Thy.12^−^, CD11b^+^ Gr1^+^, and Ly6C^med^. For monocytes and macrophages, the population was selected as CD45^low^, Thy.12^−^, Gr1^−^, CD11b^+^, and Ly6C^high^ expressing myeloid population. The samples were stained with 7-AAD and DAPI (BD Pharmingen, San Diego, CA, USA) to identify and exclude the dead population. Absolute leukocyte count was done by performing FACS analysis of the entire cell population obtained from a single mouse brain. Antibodies used for FACS experiments are as follows: anti-CD45.2 FITC (BD Bioscience, 1:100 dilution), anti-Thy1.2 APC Cy7 (Biolegend Dedham, MA, USA 1:2500 dilution), anti-Cd11b PE Cy7 (eBioscience, San Diego, CA, USA 1:2000 dilution), anti-Ly6C-PerCp Cy5 (BD Bioscience, 1:300 dilution), anti-GR1 PE (eBioscience 1:200 dilution), and anti-Ki67 PE (Biolegend, 1:250 dilution).

### 2.8. STRING Database Analysis

STRING (Search Tool for the Retrieval of Interacting Gene) is an open-source database that contains information on protein–protein interaction of 9.6 million proteins from 2031 organisms. The proteins identified through the transcriptome analysis were analyzed in STRING 10.5 for the generation of a meta-network [31]. The STRING interactions generated are based on the experimental data, computational prediction method, and referring to the public database. The output from the STRING is in the form of a network (gene-nodes and edges-interaction). The pathway analysis tool in STRING was used to determine the pathways involved. The network was further imported to the Cytoscape software for determining degree centrality analysis [32]. CytoNCA tool in Cytoscape was used for degree centrality calculation [33]. 

### 2.9. Statistical Analysis

Data are expressed as means ± SEM, and the significance of the difference between groups was evaluated by using a two-tailed Student’s *t*-test, and graphs were plotted and analyzed using the Graphpad Prism (v6.01) (Graphpad Prism, San Diego, CA, USA).

## 3. Results

### 3.1. VSV Infection of the CNS Induced Fatality and Brain Viral Load Attained Plateau at Early Stage

**Figure 1:**
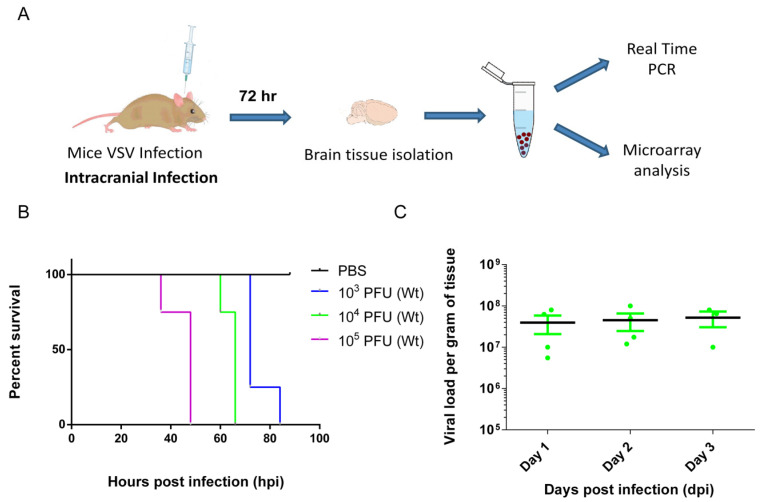
Vesicular stomatitis virus (VSV) infection in the central nervous system (CNS) induces fatal encephalitis in wild type mice. (**A**) B6 mice (*n* = 4 per group) were injected intracerebrally with the designated doses of VSV to induce encephalitis. Intracardially perfused mouse brain was collected after 72 hpi to estimate brain viral load and to collect total cellular RNA. Total cellular RNA was later used for gene microarray analysis as well as for validation of real-time PCR assays. (**B**) Kaplan–Meier survival curve of animals challenged with the varying doses of VSV (doses represented in plaque-forming units (PFU) of VSV are shown in the panel to the right of the graph). (**C**) In this experiment, all animals were intracerebrally injected with 10^4^ PFU of VSV, and brain viral loads were determined by plaque assay technique conducted in Vero cells at said time points and reported as PFU of VSV per gram of tissue.

The VSV is a Rhabdoviridae family member and is placed with other neurotropic viruses such as RV and CHPV. In the murine model, intracerebral injection of VSV causes fatal encephalitis in adult mice. Here adult B6 (C57/BL6J) mice were intracerebrally injected with varying doses of inoculum (Figure 1A). Infected mice were observed for the development of classic neurological symptoms, such as ruffled fur, convulsion, and limb paralysis, etc., and death kinetics was plotted using the Kaplan–Meier survival curve. Results show that higher viral doses (10^5^ PFU) of inoculum induced early fatality (within 48 hpi). While, lower dose (10^4^ PFU, 10^3^ PFU) administration delayed death kinetics. Nonetheless, all animals died between 40 and 84 h post-infection, which exhibited a dose-dependent pattern of death kinetics (Figure 1B). Interestingly, brain viral load kinetics in animals injected with 10^4^ PFU of VSV showed a marginal increase during the course of infection (Figure 1C). These data suggest that somehow the virus rapidly replicates in the initial stage of infection and tends to plateau later on. However, this mechanism is insufficient to prevent fatal encephalitis. As the onset of fatality was consistent at 10^4^ PFU of VSV, further animal infection experiments were done using this dose regimen.

### 3.2. The Virally Infected CNS Showed Microglial Proliferation and Influx of Peripheral Mononuclear Immune Cells

**Figure 2:**
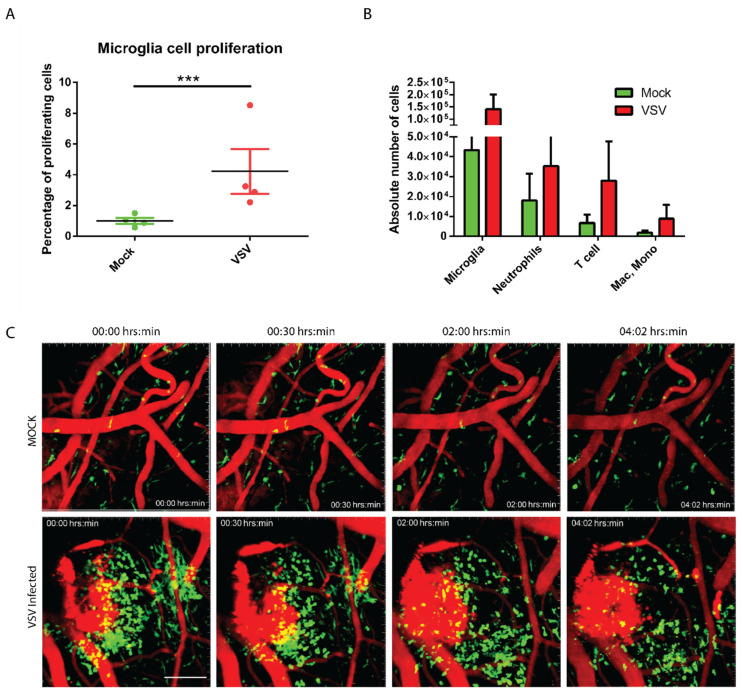
VSV infection in CNS induces microglia proliferation and myeloid cell recruitment. Mice were either infected with 10^4^ PFU of VSV or mock-infected. At day two post-infection, the total CNS myeloid cell populations were analyzed by flow cytometry. (**A**) Representative dot plot showing the percentage increase in the number of microglia (CD45^low^, Thy1.2^−^, Gr1, and CD11b^+^) population in VSV-infected mice brain (*n* = 4 per group). An asterisk denotes statistical significance, *** denotes *p* value < 0.001. (**B**) The bar graph represents the absolute count of myeloid cells obtained from mice brain (*n* = 4): microglia, neutrophils (CD45^low^, Thy.12^−^, CD11b^+^ Gr1^+^, and Ly6C^med^), monocytes, and macrophages (CD45^low^, Thy.12^−^, Gr1^−^, CD11b^+^, and Ly6C^high^) in the CNS of mice (*n* = 4 per group) following VSV infection. Note an increase in the CNS myeloid cell number in VSV-infected animals compared to the mock-infected animals (*p* > 0.05). (**C**) The two-photon light scanning microscopy (TPLSM) was performed through a surgically thinned skull window in mock-infected LysM-GFP reporter mice (upper row) and compared to VSV-infected mice at day three post-infection (lower row). Representative figure showing an increase in the number of cells infiltrated in the brain parenchyma (see corresponding movies Videos S1 and S2). Blood vessels are represented in red, and macrophages, monocyte, and neutrophils are represented in green. Please note the presence of prominent vascular leakage (damaged blood vessels in red) in the VSV-infected brain. Surrounding this, a cluster of infiltrating cells are observed at the beginning of the movie; these cells later shifted to elsewhere over a time period of 4 h.

The CNS is inhabited with an extensive network of resident immune sentinels (microglia, macrophages, dendritic cells, etc.), which are often the first responders to the infection or injury. We next performed experiments to assess the immunological status of the brain by looking at the profile of both residential as well as infiltrating myeloid cells in the infected mice brains. Flow cytometric analysis established that the microglia (CD45^low^, Thy.12^−^, Gr1^−^, and CD11b^+^), the most abundant CNS-resident myeloid cells showed evidence of proliferation (Ki-67^+^) (Figure S1). This notion is also supported by results showing an increase in the absolute number of microglia (three-fold increase) in the infected brain at day two post-infection (Figure 2A). To further assess the types of infiltrating leukocytes’ profile, we performed flow cytometric experiments. Total myeloid cell count of the infected brain revealed multiple fold increase in leukocyte counts where neutrophils showed a two-fold increase; T cells registered a six-fold increase; microglia showed a four-fold increase, macrophage and monocytes showed a three-fold increase in the number when compared to the uninfected control (Figure 2B). Additionally, using intravital two-photon microscopy imaging technique through a thinned skull window, we examined the state of myeloid cells patrolling in the virally challenged mouse brain at day three post-infection. We visualized myeloid cells using LysM^gfp/+^ reporter mice where neutrophil population appears brightest for green fluorescence relative to monocytes and macrophages. Similar to our flow cytometric data (Figure 2B), we observed an identical pattern of the influx of myelomonocytic cells in virally challenged mouse brain (Figure 2C, Supplementary Movies S1 and S2).

### 3.3. CNS Gene Expression Profile Reveals the Signature of the IFN-I Signaling Pathway

**Figure 3:**
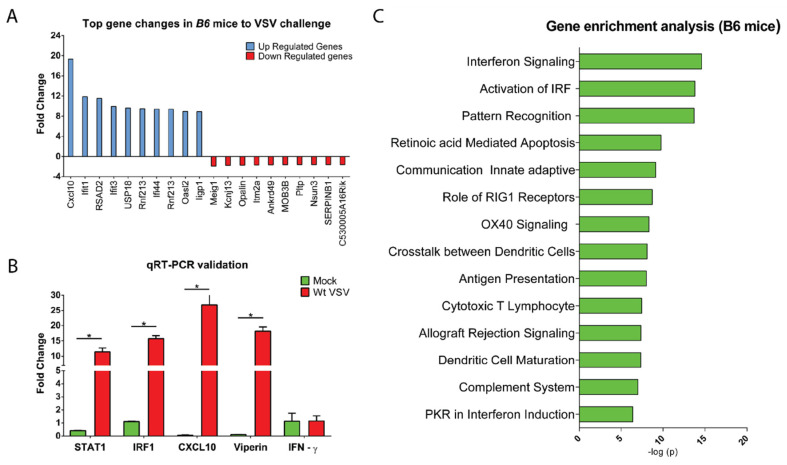
VSV infection in the brain induces IFN-I-driven antiviral response. Microarray analysis was performed on the total RNA extracted from the mice brain (*n* = 4) tissue at day three post-infection and compared to the mock-infected mice. (**A**) A representative bar graph showing the top 10 differentially expressed gene candidates (blue represent upregulation while red represent downregulation). (**B**) Few selected genes falling to the category of antiviral response (STAT1, IRF-1, CXCL10, IFN-γ, and Viperin) were validated by RT-PCR experiments. The fold change in gene expression was calculated by normalizing the data with the housekeeping gene (β-actin) as an internal control from the same sample using ∆∆Ct method. * denotes *p*-value < 0.05. (**C**) Ingenuity pathway analysis tool was used to predict the top biological canonical pathways corresponding to the gene expression profile and are represented here in the bar graph. The negative *log p* values are plotted on the graph *X*-axis.

To assess the nature of the CNS’s innate response to the viral challenge, we looked at the global gene expression profile of VSV-infected mice brains. Total cellular RNA harvested at day three post-infected mice brains were subjected to the gene microarray analysis. Out of 35,556 gene candidates input in the microarrays, 301 genes showed a statistically significant (*p* < 0.05) difference in the expression level of at least 1.5-fold change (Table S1). Further analysis (Figure 3A) showed a higher number of upregulated (*n* = 281) candidates compared to downregulated candidates (*n* = 20). We noticed an emergence of complex and diverse host factors covering cytokines, chemokines, antiviral factors, transcriptional, and translational regulatory molecules in the infected mice brains (details are in Table S1). We also validated the microarray results by real-time PCR (RT-PCR) experiment by measuring fold change expression in a few selected candidates known to have a potent antiviral response (STAT1, IRF-1, CXCL10, and Viperin, etc.) (Figure 3B). The results obtained from both transcriptome analysis as well as RT-PCR experiments showed identical expression patterns. To gain mechanistic insight into the host response associated with VSV infection, we analyzed the candidates using Ingenuity Pathway Analysis (IPA) tools and constructed canonical pathways. The top pathways showing statistical significance fell into the innate immune response category. Of these, the IFN-signaling pathway topped the list followed by activation interferon regulatory factors (2nd), pattern recognition (3rd), Retinoic acid-mediated apoptosis (4th), etc. (Figure 3C). Additionally, genes related to antigen sensing, chemoattraction, antigen presentation, and protein synthesis were highly upregulated, suggesting a strong innate response of the brain to cytopathic virus infection. Collectively, these data suggest that the CNS is immunologically active during the course viral infection, which showed a signature pattern of IFN-I signaling mediated gene expression.

### 3.4. Loss of IFN-I Signaling Rather Shifted CNS to a Robust Compensatory Mechanism to Mount an Antiviral Response

**Figure 4:**
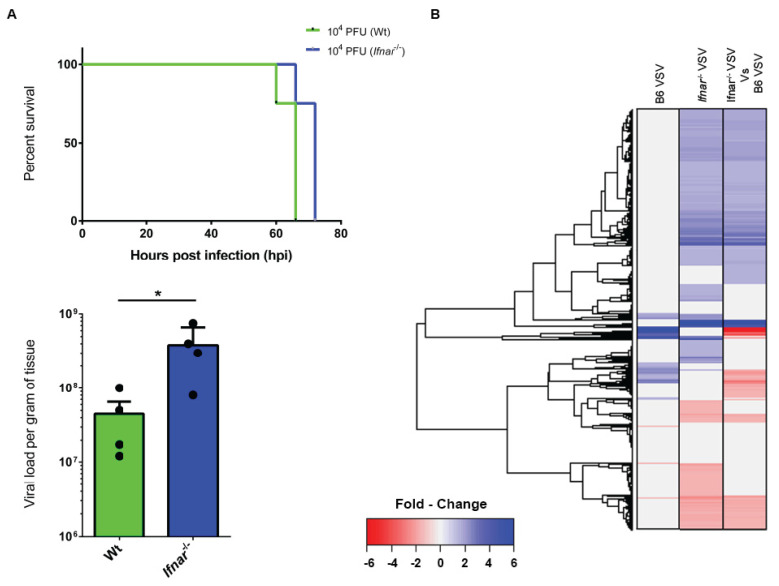
VSV infection induces a massive change in the gene expression in the absence of type I interferon. *Ifnar*^−/−^ mice (*n* = 4 per group) were intracerebrally infected with the VSV to induce encephalitis, and comparative CNS gene microarray analysis was performed along with the wild type mice. (**A**) The upper subset represents a Kaplan–Meier survival curve of animals, which shows a marginal delay in onset death in *Ifnar*^−/−^ mice. While the lower subset represents the brain viral titers determined by plaque assay, which shows a significant increase (*p* < 0.05) in brain viral load in *Ifnar*^−/−^ mice. * denotes *p*-value < 0.05 (**B**) Global heat map depicting the change in gene expression patterns and clustered based on functional relatedness (see corresponding Table S1). The change in expression level is depicted by blue (upregulation pattern), red (downregulation pattern), and gray (no change in the pattern).

Type I interferons are the early secretory molecules in a viral infection, which set the stage for mounting a further antiviral response. As our gene microarray results revealed a central role of IFN-I signaling, we asked the question of how does the brain respond to viral challenges in the absence of IFN-I signaling? To better understand the contribution of IFN-I signaling, we next measured viral load in the mice lacking the receptor for IFN-I (*Ifnar*^−/−^). As expected, we noticed a nearly eight-fold (*p* = 0.04) increase in viral load in *Ifnar*^−/−^ mice brain (Figure 4A). The *Ifnar*^−/−^ mice showed a marginal delay (6 h) in the onset of death (*p* = 0.03) (Figure 4A). However, there was no apparent distinction in the timing of the onset of neurological manifestations. At the global gene expression level, to our surprise, we noticed a significant increase in the number of gene expression in the absence of IFN-I signaling. Overall, 1357 candidates showed upregulation, while 564 candidates showed a downregulation profile in *Ifnar*^−/−^ mice (Table S1 and Figure S3). To better illustrate the comparative gene expression profile, we constructed a combined focused heat map analysis by comparing both experimental groups (Wt and *Ifnar*^−/−^ mice). This analysis revealed a distinct pattern of gene expression clustered into distinct subsets based on functional relatedness (Figure 4B).

**Figure 5:**
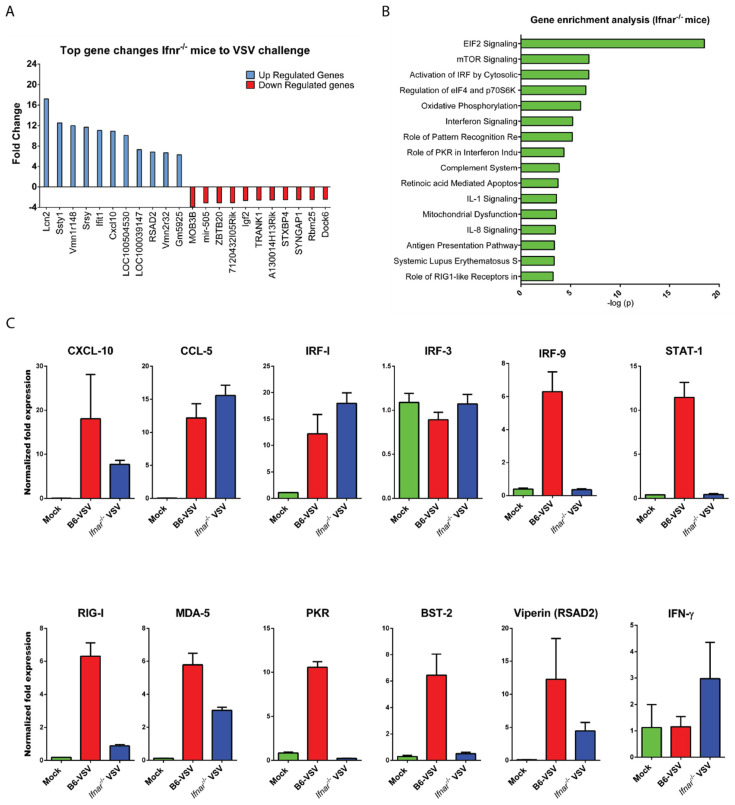
VSV infection in the brain induces type I IFN signaling-independent innate immune pathways. Microarray analysis was performed on the total RNA extracted from the mice brains at day three post-infection along with mock-infected control. (**A**) A representative bar graph showing the top 10 candidates differentially (upregulated in blue and downregulated gene in red). (**B**) Ingenuity pathway analysis tool was used to find the top biological pathway in the VSV-infected mice brains and are represented in the bar graph. The negative log *p* values are plotted on the graph *X*-axis. (**C**) A few selected genes were subjected to qRT-PCR analysis to validate the temporal change in the expression observed in microarray analysis, e.g., CXCL10, CCL-5 IRFs, BST-2, Viperin, etc. The fold change in these genes was calculated by normalizing the data with housekeeping gene (β-actin) as an internal control from the same sample using ∆∆Ct method.

Interestingly, despite the loss of IFN-I signaling, a large number of ISGs showed a significantly higher level of expression in *Ifnar*^−/−^ mice to VSV challenge (Table S1 and Figure 5A). Some of these include potent antiviral factors such as lipocalin2, CXCL10, CCL5, Viperin, Ifih1, MDA5, etc. The IPA analysis for the canonical pathways revealed a noticeable change in the signaling events in *Ifnar*^−/−^ mice. Among these, EiF2 signaling (1st), mTOR signaling (2nd), and activation of IRF by cytosolic pattern recognition receptors pathways (3rd) topped the list (Figure 5B). Surprisingly, three pathways linked to IFN signaling also remained in the top 10 categories. We also verified the microarray results by real-time PCR by picking various candidates falling into the category of cytokines (CXCL10, CCL5), regulatory factors (IRF7, IRF9, IRF3), innate sensing (RIG-I, MDA5), and antiviral genes (BST-2, IFNγ, PKR) (Figure 5C). All the RT-PCR results corroborated the findings of microarray data.

**Figure 6:**
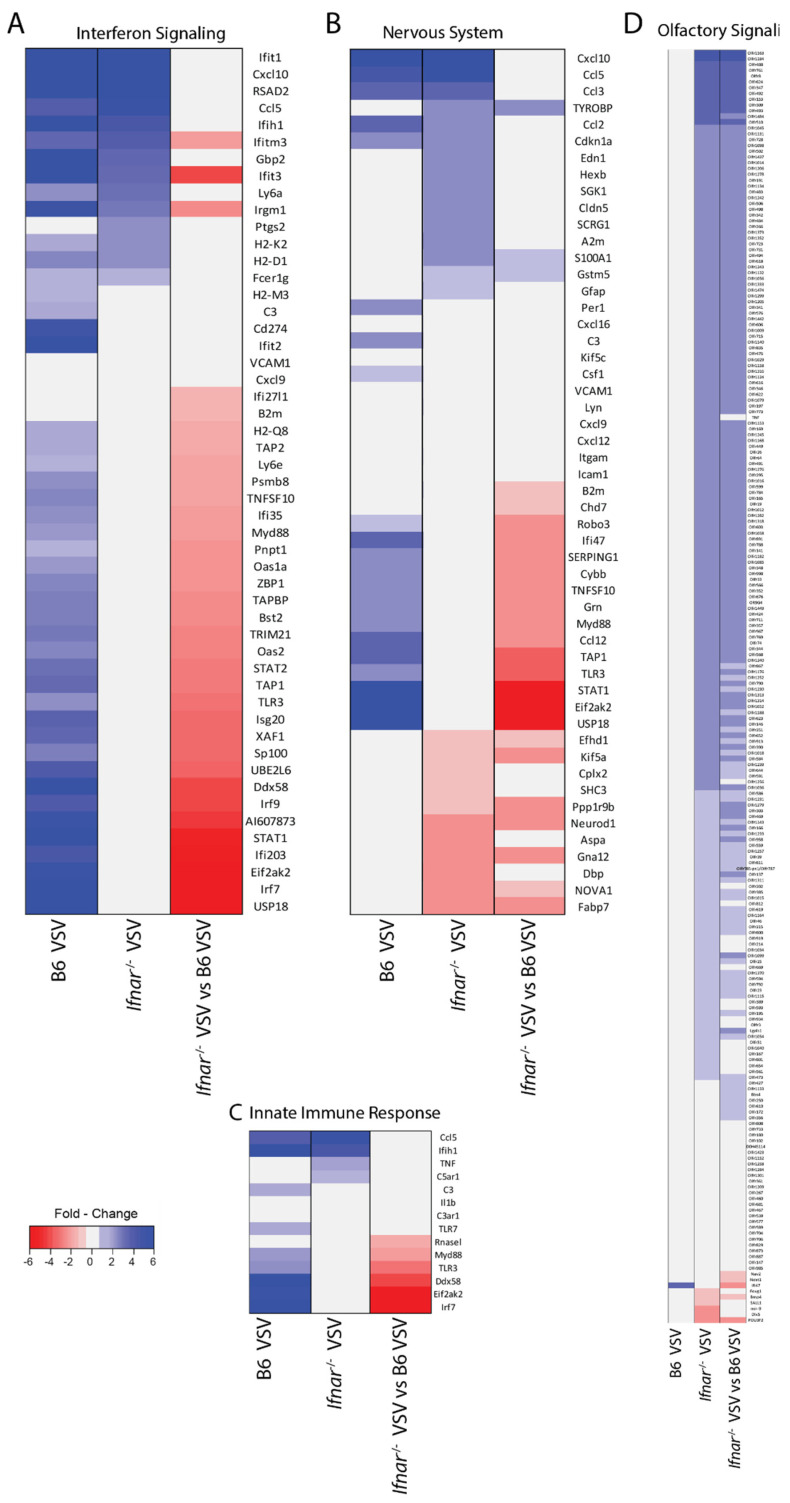
Massive gene expression in the absence of IFN-I signaling during VSV infection. Representative heat maps are shown to differentially regulate genes (*p* < 0.05, fold expression >1.5) in the absence of the IFN-I signaling associated with the (**A**) IFN signaling, (**B**), nervous system, (**C**), innate immune response, and (**D**) olfactory related signaling genes at day two post-infection. The aforementioned function was assigned to a gene using the Ingenuity Pathway Analysis (IPA) analysis tool. Blue nodes = upregulated genes, red nodes = downregulated genes, gray nodes = no change.

Next, we dissected the gene expression pattern in Ifnar^−/−^ mice by grouping the candidates into major innate immune response pathways. Combined heatmap analysis of four significant pathways showed distinct gene expression patterns in the groups associated with INF signaling (Figure 6A) and pattern recognition pathway (Figure 6C). On the other hand, a plethora of new sets of genes showed a higher expression profile in *Ifnar*^−/−^ mice affecting critical cognitive functions such as nervous system function (Figure 6B) and olfactory functions (Figure 6D) were found to be upregulated in *Ifnar*^−/−^ mice. In *Ifnar*^−/−^ mice, the Lipocalin 2 (LCN2) topped the list of upregulated genes followed by interferon-induced protein with tetratricopeptide repeats 1B (IFIT1B). Among the chemokine’s family members, *Ifnar*^−/−^ mice showed a lower fold change in CXCL10 expression as compared to its wild type counterpart. However, CCL5 expression was higher (eight-fold) in *Ifnar*^−/−^ mice compared (five-fold) to wild type (Figure 5C). Elevated expression of CCL3, which is known to augment CD8+ T cell effector function and migration was observed in the *Ifnar*^−/−^ mice (Table S1). Among the regulatory transcription factors, loss of IFN-I signaling resulted in the complete abrogation of IRF3, IRF7, IRF9 expression, while IRF1 showed increased expression profile in *Ifnar*^−/−^ mice in response to VSV challenge. We also noticed the elevated expression of antiviral genes such as viperin, IFN-γ, RIG-I, and MDA5, etc., those critical regulators in the innate immune response. Except IRF-1 and CCL-5, the relative fold change level was observed lower in *Ifnar*^−/−^ mice compared to Wt (Figure 5C). Thus, IFN-I signaling loss is compensated to a large extent by activation of alternate pathways to drive immune response. We also noticed the downregulation of a few candidates. These include Zinc finger and BTB domain-containing 20 (ZBTB20), a member of BTB/POZ family. Previous studies have shown that ZBTB20 could promote TLR-mediated antiviral response [34]. Other candidates include MOB3B (MOB Kinase Activator 3B), insulin growth factor-2 (IGF2), Tetratricopeptide repeat, and ankyrin repeat-containing 1 (TRNK1). Among these IGF2 is a growth promoter, anti-apoptotic in nature, and associated with virus-induced carcinogenesis [35]. Downregulation of IGF2 could, therefore, further enhance VSV-induced apoptosis.

### 3.5. Bioinformatics and Network Analysis Suggest the Central Role of TNF in the Antiviral Immune Response

**Figure 7:**
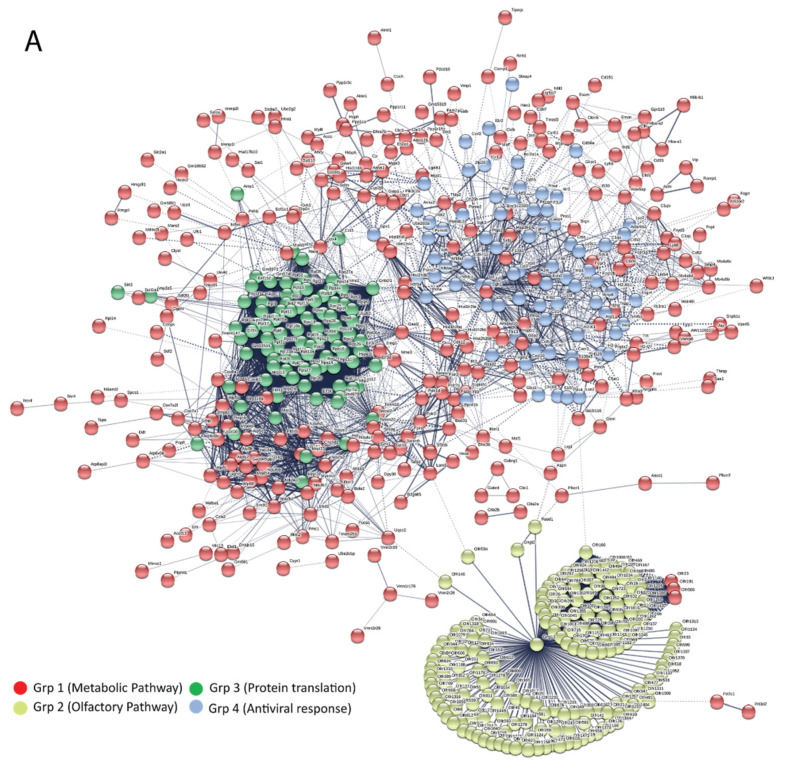

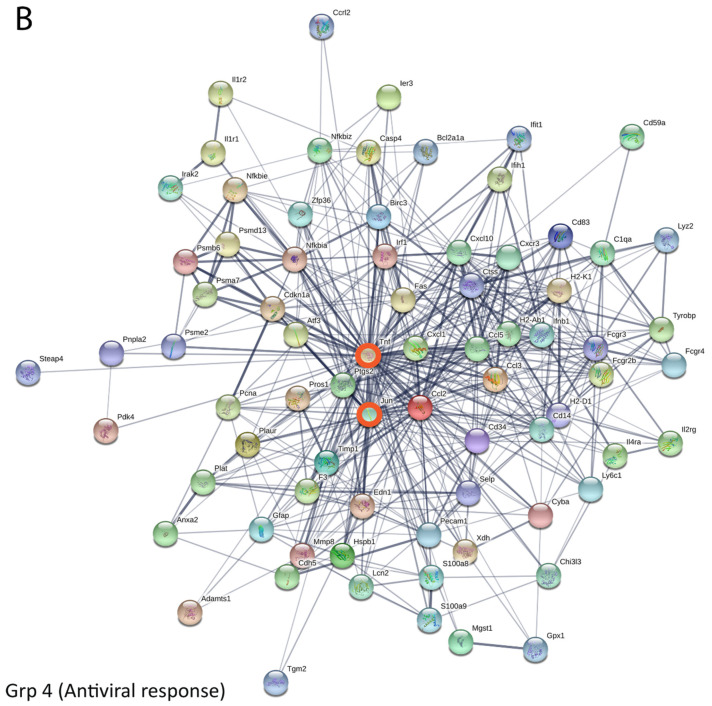
Network analysis of the up-regulated gene in *Ifnar*^−/−^ mice reveals a TNF to be a crucial regulator for antiviral immune response. The figure depicts a modular network formed using the upregulated genes. (**A**) protein-to-protein interaction (PPI) network was developed using the STRING (Search Tool for the Retrieval of Interacting Gene) database. The interaction was divided into four groups using the K-means clustering tool from the STRING database. Each group defines the essential unique function: group 1 includes metabolic pathway genes, group 2 comprises olfactory related gene, group 3 represents the protein translation gene, and group 4 contains antiviral response-related genes. (**B**) Later, group 4 genes representing the antiviral response were used for the interactome generation by using the STRING database. The network shows the maximum connectivity patterns where Jun was found to have the highest number of connective nodes, followed by that of TNF-α.

To identify the critical host proteins and pathways involved in the IFN-I-independent activation of the ISGs, network analysis, and Kyoto Encyclopedia of Genes and Genomes (KEGG) pathway analysis were performed by using the Search Tool for the Retrieval of Interacting Genes/Proteins (STRING) 10.5 online database (https://string-db.org/). A protein–protein interaction network diagram was constructed for differentially regulated genes associated with *Ifnar*^−/−^ infected mice (Figure 7A). When the network was subjected to gene clustering analysis (K clustering), four distinct groups emerged. Further, the KEGG enrichment analysis was performed to assign a functional basis to these groups. This analysis revealed that group-1 genes were associated with metabolic pathways, group-2 with olfactory function pathways, group-3 with protein translation, and group-4 with antiviral immune response functions. The vast majority of upregulated genes were mostly involved in olfactory functions, followed by ribosomal signaling pathways (Table 1).

Table 1. KEGG pathway analysis for the differentially regulated gene during VSV infection. Gene enrichment analysis was performed on all upregulated gene candidates in the VSV-infected *Ifnar*^−/−^ mice. Table S1 provides the information on 1357 upregulated genes acquired from the microarray analysis that were divided into four groups using STRING database and were used for the KEGG pathway analysis. False discovery rate and gene number count was used as a parameter to identify a pathway involved.

We then performed a detailed analysis of group-4 genes (antiviral immune response). The STRING database analysis (Figure 7B) revealed that Jun and TNF showed a maximum interactome profile among the upregulated candidates. The network score was further imported into the Cytoscape software, and the degree centrality analysis was performed using the CytoNCA tool of Cytoscape with Combine score from STRING database as an edge weight. The degree centrality analysis revealed the TNF signaling as the topmost activated and influential pathway indicating its central role in orchestrating antiviral immune response in *Ifnar*^−/−^ mice (Figure 7B and Table 2). These data suggest the presence of MDA5 (encoded by IFIH1 gene) associated with activation of IRF1 might be a key factor in TNF-driven inflammatory response. Collectively, our analysis suggests that elevated expression of TNF, along with IFN-γ, could induce ISGs expression and antiviral innate immune response in the absence of IFN-I signaling. Thus, indicating that TNF and IFN-γ mediated signaling in part compensate for IFN-I signaling loss driving antiviral immune response to VSV infection in *Ifnar*^−/−^ mice.

Table 2. Degree centrality analysis of antiviral response gene (group 4) revealing the top 10 candidates. Antiviral response-related genes, identified during the generation of the interactome, were uploaded in Cytoscape to calculate the degree centrality using the CytoNCA tool. The combined score of interaction from STRING database was used as an edge weight for the degree centrality calculation.

## 4. Discussion

While the anatomical structures such as BBB and blood-CSF barrier, etc., effectively protect the CNS against invading pathogens, the brain homeostasis is primarily maintained by the resident immune cells. Upon the viral entry, the CNS innate immune mechanisms often respond appropriately to contain the pathogen until the arrival of adaptive immune cells. An increasing number of studies highlight that the quality of the innate response directly influences the overall outcome of CNS pathogenesis mediated through an adaptive response. While studying the innate response to cytopathic virus infection, we uncovered that though type I IFN signaling plays a central role, its absence can be compensated by robust mechanisms to act upon on viral challenge. In comparison to our previous observation on a non-cytopathic virus infection (LCMV) where IFN-I signaling loss abrogated almost all of the CNS gene expression [20], the VSV infection in *Ifnar*^−/−^ mice instead triggered a profound and differential pattern of gene expression. Unlike the earlier observation of *Ifnar*^−/−^ mice becoming an asymptomatic and lifelong carrier of LCMV, the VSV infection instead induced slightly late fatality associated with an increased viral load in the brain. As VSV is highly sensitive to IFN-I, a higher viral load is expected in *Ifnar*^−/−^ mice. The marginal delay in death kinetics in *Ifnar*^−/−^ might be due to the reduced cytokine-driven proinflammatory response and associated immunopathology. However, it is quite unexpected to observe a dramatic increase in the number of gene changes (*n* = 301 in wt vs. *n* = 1921 in *Ifnar*^−/−^, Figure S2). The surprisingly higher number (*n* = 220) of dysregulated genes were associated with olfactory functions, which were silent in the presence of IFN-I signaling. A study conducted by modelling intranasal infection of VSV has documented that the virus tends to reside in the glomerular structure of the olfactory bulb. Additionally, in the absence of local IFN-I response, the virus gets the upper hand in reaching much higher titer in infected mouse brain [36]. However, in normal mice, intranasal infection typically restricts the virus in the olfactory lobe and gets resolved due to effective T cell response. Another study by the Van den Pol group has documented the connection between VSV-induced ISG expression and the spread of viruses in olfactory neurons [37]. Considering all these observations, although we are not explaining the exact reason behind this massive change in olfactory signaling, we speculate that higher viral load in *Ifnar*^−/−^ mice might have resulted in a change in the spatiotemporal distribution of the virus spreading to the olfactory bulb of the brain. This spread might have been facilitated in the absence of IFNAR signaling-driven antiviral restriction factors [36].

Another explanation of *Ifnar*^−/−^ mice responding differently to VSV compared to non-cytopathic virus infection is due to the nature of the viral life cycle. The VSV has faster growth kinetics, and we noticed this reached its near-peak level, just one day after infection (Figure 1C). Although, it is possible that the viral load reaching an early plateau might have resulted from a relatively higher dose (10^4^ PFU) of inoculum. Further, VSV is a rapid inducer of apoptosis in neuronal populations resulting in the accumulation of dying cells in brain tissues. Under this situation, microglia being the resident macrophage-like cells, could be activated for clearance of dying cells. Earlier, we documented that in response to neuronal injury, microglia transform to activated phenotypes in just a few minutes [38]. Hence, an increase in glial populations with a higher percentage of cells showing proliferative markers (Figure 2A,B) could be a manifestation of appropriate innate responses to resolve extensive neuronal damage.

The IFN-I is known to trigger microglial proliferation [20,39]. The higher level of proinflammatory cytokines such as CXCL10 (primarily produced by neuronal and glial populations), CCL5, CCL2, etc., are known to mediate leukocyte influx and their effect is well supported by the increase vascular patrolling of neutrophils observed during intravital imaging. Interestingly, loss of IFN-I signaling resulted in further elevated expression of CCL5 (probably as a compensatory mechanism). Studies conducted with WNV virus infection also shows that increase in cytokine and chemokine response to be part of CNS pathogenesis [26]. IFN-I-independent expression of CXCL-10 in the case of HIV-I infection is shown previously, and our study is in-line with these observations [40]. Similarly, with hepatitis C virus (HCV) infection, IFN-I independent expression of CXCL10 is executed by the combinatorial action of IRF3 and NF-κB [41]. Here, we observed that in *Ifnar*^−/−^, there is a marginal increase in IRF3 expression, while IRF1 expression was significantly higher than the wild-type counterpart.

During the viral infection, three types of IFNs, e.g., IFNα, IFN-β, and IFN-γ (based on their receptor preference), take the initiative for an antiviral response. The IFN-Is are produced mainly in response to stimuli from the pattern recognition receptors (PRRs). In the context of RNA virus infection (e.g., VSV), cytosolic PRRs such as TLR-3, RIG-I, MDA5, etc., often recognize viral nucleic acids and activate IRFs, MAP kinase, and NF-κB pathways to induce antiviral responses through the expression of innate immune genes. The IFN-β promoter has a binding site for the activator protein 1 (AP1), NF-κB, and IRF transcription factors. Thus, activation of IFN-β triggers an autocrine loop by binding to its cognate receptor and activating Jak/STAT-dependent expression of ISGs. Many cytokines are a potent activator of NF-kB and AP1 [42,43,44]. The IFN-γ being a potent activator the Jak/STAT signaling associated with pro-inflammatory, pro-apoptotic, and antiviral pathways, an elevated level of IFN-γ in the *Ifnar*^−/−^ mice, explains at least in part the compensatory mechanism against IFN-I signaling loss. IFN-γ alone or in combination with TNF is known to induce sustained ISGs expression through the IRF1 pathway. In our case, elevated IRF1 and IFN-γ indicates the underlying mechanism of IFN-γ driven response. The explanation of noticeable amount IFN-I signaling pathway-linked gene expression in *Ifnar*^−/−^ mice might be due signaling cascade initiated by cytoplasmic RNA sensors such as MDA5 signaling and to lesser extent by RIG-I. Earlier studies with Sendai virus, a negative-sense RNA enveloped RNA virus infection, showed an increase in MDA5 expression independent of IFN-I (and STAT-I) signaling in the cells having constitutive activation of IRF3 [45]. In this study, we did not notice much change in IRF3 expression in Wt or Ifnar^−/−^ mice. However, knowing the activation status (phosphorylation and dimerization) of IRF3 would certainly be of help in understanding the underlying mechanism.

Further, in many viral infections, TNF-α and IFN-I are the essential effector molecules that drive the innate immune response where TNF-α works in synergy with IFN-I. However, TNF-α alone can play a potential protective role against the pathogen like *Cryptococcus neoformans* [46,47], *Mycobacterium tuberculosis* [48], VSV [49], encephalomyocarditis virus [50], HSV [51], influenza virus [52], and hepatitis B virus [53], although this phenomenon is not universal. In contrary to the antiviral role, TNF-α supports hepatitis C virus propagation [54,55]. In Ifnar^−/−^ mice microarray and RT-PCR data on the elevated expression of TNF-α (1.92-fold), IFN-β (two-fold increase), and IRF1 (three-fold increase) further suggest the existence of a compensatory mechanism to counter VSV infection in the absence of IFN-I signaling. We also speculate that local TNF-α expression is associated with myeloid cell influx to the CNS by compromising the BBB integrity.

The recombinant VSV-based platform is currently being explored extensively for vaccine development and oncolytic virotherapy. Therefore, any shortcomings concerning the neurotropic nature of the virus must be addressed diligently, especially in immune-suppressed patients. Our observation would provide a guiding principle for the therapeutic use of recombinant VSV. An extensive understanding of the mechanism underlying the high neuropathology and innate immune response of CNS during VSV infection is highly desirable. Here, we showed that in the absence of interferon signaling pathway, TNF-α and IFN-γ might play a crucial role and activate the ISG expression that could activate expression of antiviral factors such as viperin (RSAD2) and Interferon induced with helicase C domain I (IFIH1) during the early phase of infection. Nonetheless, none of these mechanisms appear sufficient to contain VSV spread and prevent a fatality. Instead, it exacerbates neuropathology. Moreover, during VSV infection, perhaps due to the release of death associated molecular patterns (DAMPs), the nature of the immune response is getting shifted to a high state alert showing higher gene expression patterns.

## 5. Conclusions

The CNS is prone to attack by a wide range of pathogens including a number of neurotrophic viruses. Experimental animal model-based studies are being employed to understand the mechanism of CNS pathogenesis and uncover the immunological and virological aspects of host–pathogen interactions. While, great progress is achieved in identifying etiology of tissue damage, neurological dysfunctions, etc., many other aspects covering brain tissue repair, signaling events underlying immunopathology, triggering signals of death cascade, etc., are not fully understood. In this study, using an experimental encephalitis mouse model, we looked at broader aspects of the nature of innate response to cytopathic viral challenge.

In contrast to non-cytopathic virus infection, our study suggests that the CNS innate response mechanism has a relatively higher-level synergy between MDA5, IRF1, CCL5, TNF-α, and IFN-γ to compensate for the IFN-I signaling loss to deal with a cytopathic virus infection. Modulation of these signaling molecules might bring positive outcomes and could attenuate degree of CNS inflammatory response in host favor. Though our study expands the current understanding around IFN-I signaling, further detailed investigations are required to comprehend the specific signaling events associated with this phenomenon. Our study is limited with experimentation with Ifnar^−/−^ mice. It would be more appropriate to expand these observations to Ifnar^−/−^ and TNF-alpha receptor double knock-out mice for studying the VSV challenge. Similarly, experimenting with IFN-γ receptor knock-out (GRKO) would further help in unraveling the mechanism of this phenomenon. Nonetheless, our study provides background knowledge about the presence of compensatory innate immune pathways in the CNS and could help in developing a therapeutic intervention against viral encephalitis.

## Figures and Tables

**Table 1:** Kyoto Encyclopedia of Genes and Genomes (KEGG) pathway analysis for the differentially regulated gene during VSV infection.

Pathway ID	Pathway Description	Observed Gene Count	False Discovery Rate	Group
190	Oxidative phosphorylation	21	6.45 × 10^−11^	Group 1
5012	Parkinson’s disease	19	1.06 × 10^−8^	
5016	Huntington’s disease	20	5.89 × 10^−8^	
5010	Alzheimer’s disease	19	8.50 × 10^−8^	
4932	Non-alcoholic fatty liver disease (NAFLD)	17	6.37 × 10^−7^	
1100	Metabolic pathways	42	3.44 × 10^−2^	
4260	Cardiac muscle contraction	7	3.58 × 10^−2^	
4740	Olfactory transduction	175	1.55 × 10^−229^	Group 2
3010	Ribosome	39	4.78 × 10^−64^	Group 3
4668	TNF signaling pathway	11	2.14 × 10^−11^	Group 4
5168	Herpes simplex infection	12	3.38 × 10^−10^	
4380	Osteoclast differentiation	10	9.39 × 10^−10^	
4060	Cytokine–cytokine receptor interaction	12	2.32 × 10^−9^	
5140	Leishmaniasis	8	2.36 × 10^−9^	
5142	Chagas disease (American trypanosomiasis)	9	2.77 × 10^−9^	
5164	Influenza A	10	9.71 × 10^−9^	
5166	HTLV-I infection	11	4.71 × 10^−8^	
4620	Toll-like receptor signaling pathway	8	5.01 × 10^−8^	
4145	Phagosome	9	9.90 × 10^−8^	
5152	Tuberculosis	9	1.45 × 10^−7^	
4640	Hematopoietic cell lineage	7	2.64 × 10^−7^	
5150	Staphylococcus aureus infection	6	3.59 × 10^−7^	
4064	NF-kappa B signaling pathway	7	4.36 × 10^−7^	
4621	NOD-like receptor signaling pathway	6	6.97 × 10^−7^	
4650	Natural killer cell-mediated cytotoxicity	7	1.85 × 10^−6^	
4612	Antigen processinu67t7uygtb hn nng and presentation	6	2.54 × 10^−6^	
4610	Complement and coagulation cascades	6	3.33 × 10^−6^	
4210	Apoptosis	6	4.39 × 10^−6^	

**Table 2:** Degree centrality analysis of antiviral response gene (group 4) revealing the top 10 candidates.

Gene Name	Degree Centrality (Weight)
Tnf	50.06092
Ccl2	25.065063
Jun	24.664934
Ccl5	18.67208
Cxcl10	17.798105
Ptgs2	15.644774
Nfkbia	14.423865
Cd14	13.918694
Irf1	13.681601
Fcgr3	13.137255

## Supplementary Materials

The following are available online at https://www.mdpi.com/2076-393X/8/2/326/s1, Figure S1: CNS Myeloid cells proliferated during VSV infection. Expression of the Ki67, a proliferative marker, on the microglia (CD45^low^, Thy.12^−^, Gr1^−^, and CD11b^+^) resident in the CNS is shown in the figure, Figure S2: VSV infection of the IFNR−/− mice brain drives massive changes in cellular gene expression. Mice were either infected with 10^4^, PFU or VSV/mock-infected by the intracranial route. At day three post-infection, the brains were harvested, and total RNA was used for gene microarray analysis. The graph shows the total number of up and downregulated genes in the presence and absence of IFN signaling, Table S1: Differential expression of genes during VSV infection of CNS. The table here shows the microarray analysis data that was performed on the RNA extracted from the brain of VSV-infected wt mice and Ifnar^−/−^ mice at day three post-infection. The data includes 2658 genes that were differentially regulated during CNS infection with VSV. Positive numbers indicate an increase in expression, whereas, negative numbers represent decreased expression (relative to mock), Table S2: QPCR primer list. A list of primers, along with the NCBI gene identification number, is provided that was used for the RT-qPCR. (DOC), Video S1: Homing of myeloid cells in uninfected mouse brain. Representative time-lapses and 3D reconstructed movie of myeloid cells (green) captured by TPLSM through a thinned skull in the mock infected mice. Quantum dots are used for blood vessel labeling (red). The image was captured for 4 h after three-day post mock infection. This movie shows homing of neutrophil/macrophages in the CNS in normal condition. Video S2: Infiltration of innate myeloid cells and CNS patrolling during the VSV infection. Representative time-lapses and 3D reconstructed movie of myeloid cells (green) captured by TPLSM as mentioned in Video S1. The image was captured for 4 h after three-day post VSV infection. This movie shows the recruitment of the neutrophil/macrophages in the CNS after VSV Infection. Please note significant vascular leakage is observed in the VSV-infected brain and surrounding this a cluster of myeloid cells appear at the beginning of the movie. Over time, these cells shifted elsewhere. (MOV) Movie.
