# Supplementary material for: IFN-I Independent Antiviral Immune Response to Vesicular Stomatitis Virus Challenge in Mouse Brain
Source: Vaccines (Basel). 2020 Jun 19;8(2):326. doi: 10.3390/vaccines8020326 (PMC7350232; doi:10.3390/vaccines8020326)
Supplement: Supplementary file 1 [file vaccines-08-00326-s001.zip › Supplementary data/Table S2.docx]

**Table S2. QPCR Primer List**

| **Gene** | **NCBI ID** | **Forward primer** | **Reverse primer** |
| --- | --- | --- | --- |
| Actin | NM_007393 | AGCTCATTGTAGAAGGTGTGG | GTGGGAATGGGTCAGAAGG |
| Bst-2 | NM_198095 | CTCTTTCTATCACTATCTGCCCG | GGCGAAGTAGATTGTCAGGATG |
| CCL5 | NM_013653 | GGGTACCATGAAGATCTCTGC | TCTAGGGAGAGGTAGGCAAAG |
| CXCL10 | NM_021274 | TCAGCACCATGAACCCAA | CTATGGCCCTCATTCTCACTG |
| IFN γ | NM_008337 | TCAAGTGGCATAGATGTGGAA | TGGCTCTGCAGGATTTTCATG |
| IRF-1 | NM_008390 | GAAGGGAAGATAGCCGAAGAC | TCTGGTTCCTCTTTGCAGC |
| IRF-3 | NM_016849 | CACAAGGACAAGGACGGAG | ATGCAGAACCACAGAGTGTAG |
| IRF-7 | NM_016850 | TTGATCCGCATAAGGTGTACG | TTCCCTATTTTCCGTGGCTG |
| IRF-9 | NM_008394 | CTCTTTGTTCAGCGCCTTTG | GTACTGGGCCAAATCTCTACAG |
| MDA -5 | NM_027835 | GGTGGACAAACTTCTGATTAACG | TCCTTCTGCACAATCCTTCTC |
| PKR | NM_011163 | TGGCTTAGGTGGATTTGGTC | GTTGACGTGATTGAGTTCTGC |
| RIG-I | NM_172689 | GATGAAGGAGACAGAGAAGCTAG | TCTGCCATCTGAAACACTGA |
| STAT1 | NM_009283 | GATCTCCAACGTCAGCCAG | GAAAACTGCCAACTCAACACC |
| Viperin | NM_021384 | GAAACATTCTTGGAGCGTCA | CAGAATAGACTTGGAAGGGTCC |
