# Supplementary figures and images for: IFN-I Independent Antiviral Immune Response to Vesicular Stomatitis Virus Challenge in Mouse Brain
Source: Vaccines (Basel). 2020 Jun 19;8(2):326. doi: 10.3390/vaccines8020326 (PMC7350232; doi:10.3390/vaccines8020326)

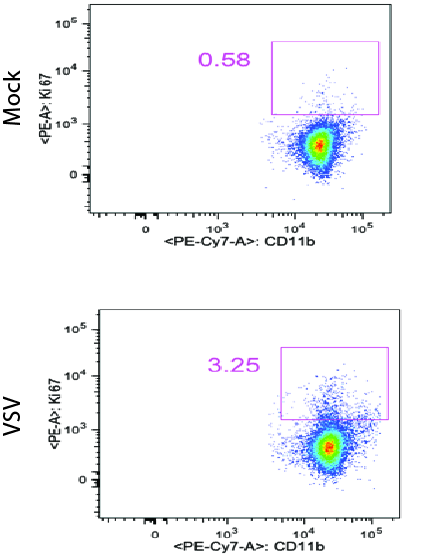

Supplement: Supplementary file 1 [file vaccines-08-00326-s001.zip › Supplementary data/Supplementary Figure S1.tif]

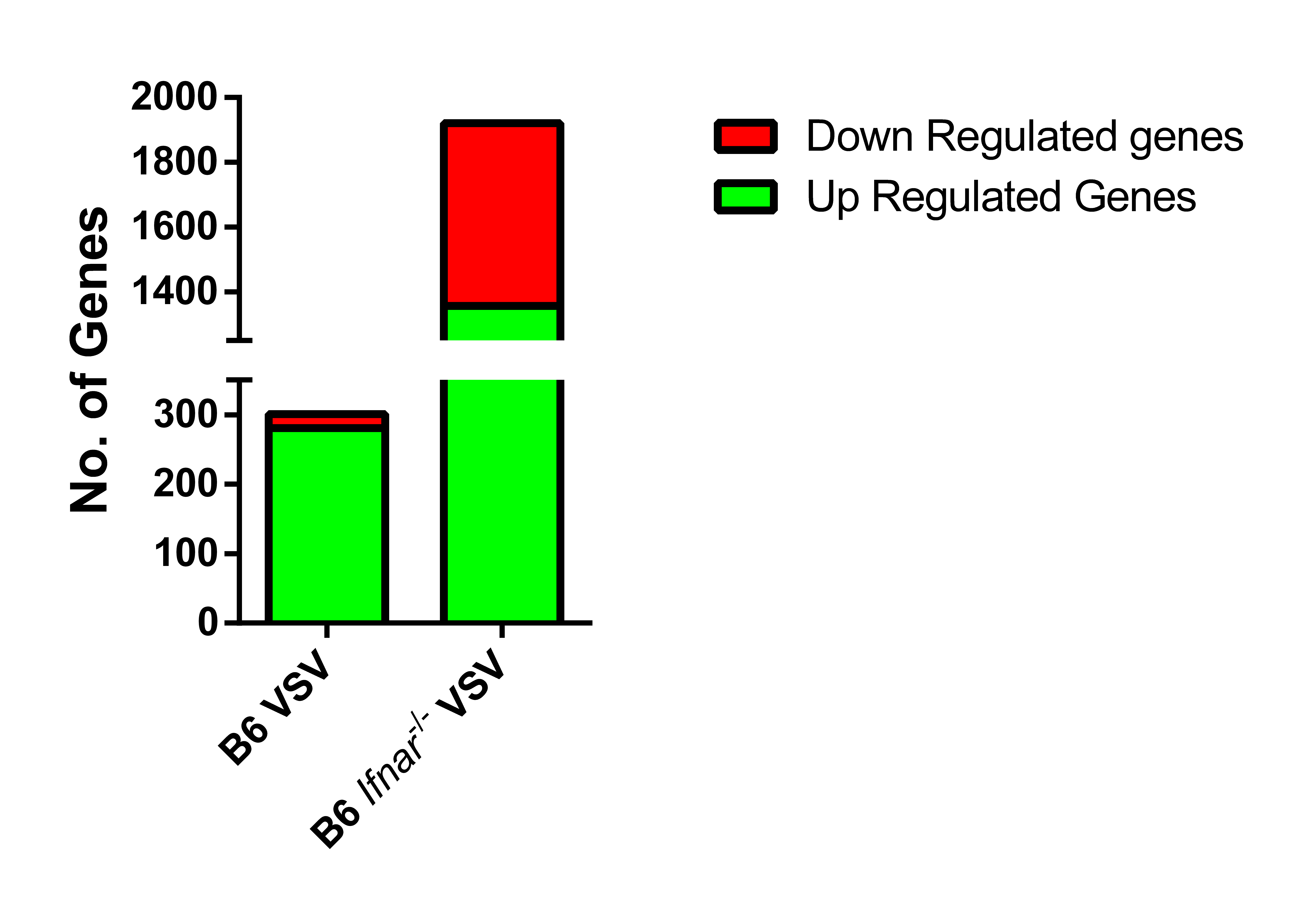

Supplement: Supplementary file 1 [file vaccines-08-00326-s001.zip › Supplementary data/Supplementary Figure S2.png]
